# Estimating blue mussel (*Mytilus edulis*) connectivity and settlement capacity in mid-latitude fjord regions

**DOI:** 10.1038/s42003-023-05498-3

**Published:** 2024-02-09

**Authors:** Ana Corrochano-Fraile, Stefano Carboni, Darren M. Green, John B. Taggart, Thomas P. Adams, Dmitry Aleynik, Michaël Bekaert

**Affiliations:** 1 Institute of Aquaculture, Faculty of Natural Sciences, University of Stirling, Stirling, UK; 2 Fondazione IMC, Torre Grande, Oristano, Italy; 3 Scottish Sea Farms Limited, Barcaldine Hatchery, Argyll, UK; 4 Scottish Association for Marine Science, Oban, UK

**Keywords:** Biophysics, Genetics, Computational biophysics, Population dynamics, Population genetics

## Abstract

The mussel industry faces challenges such as low and inconsistent levels of larvae settlement and poor-quality spat, leading to variable production. However, mussel farming remains a vital sustainable and environmentally responsible method for producing protein, fostering ecological responsibility in the aquaculture sector. We investigate the population connectivity and larval dispersion of blue mussels (*Mytilus edulis*) in Scottish waters, as a case study, using a multidisciplinary approach that combined genetic data and particle modelling. This research allows us to develop a thorough understanding of blue mussel population dynamics in mid-latitude fjord regions, to infer gene-flow patterns, and to estimate population divergence. Our findings reveal a primary south-to-north particle transport direction and the presence of five genetic clusters. We discover a significant and continuous genetic material exchange among populations within the study area, with our biophysical model’s outcomes aligning with our genetic observations. Additionally, our model reveals a robust connection between the southwest coast and the rest of the west coast. This study will guide the preservation of mussel farming regions, ensuring sustainable populations that contribute to marine ecosystem health and resilience.

## Abstract (web summary)

A study of mussel populations in Scottish waters found that mussel larvae move from south to north and form five distinct genetic groups. This helps preserve mussel farms and marine ecosystems.

## Introduction

Over the past two decades, the blue mussel industry in mid-latitude fjord environments has faced significant challenges, marked by fluctuations in production^1^ caused by low levels of spat settlement and poor-quality spat. Certain regions along the western continental coasts, like Scotland, have also encountered production losses due to issues such as fragile shells and low-quality meat^2–4^. However, despite these obstacles, the industry has shown remarkable growth, with the output of mussels produced surging by 52% from 5661 tonnes in 2020 to a record-breaking 8590 tonnes in 2021^1^. This remarkable increase in production not only signifies the highest level of mussel production ever recorded in Scotland but also underscores the expanding nature of the mussel farming sector in the region. Mussel farming in Scotland is primarily conducted through an off-bottom approach, where ropes are utilised to cultivate the mussels^5^. The main producers are strategically situated along the west coast of Scotland, near the shore, in dynamic areas. The recruitment process for mussels involves both natural spawning from wild populations and seeding from existing mussel farms^6,7^.

Addressing fluctuations in mussel production is crucial: Mussels are a vital component of aquatic ecosystems, serving as keystone species and providing numerous ecosystem services such as water filtration, habitat creation, coastal defence, carbon sequestration, and nutrient cycling^8^. Moreover, blue mussel aquaculture is both an eco-friendly protein source and a crucial component of the seafood industry in Western Europe and Scotland, with the added benefit of being used as sustainable feed for farmed fish, promoting environmental responsibility within the aquaculture sector^1,9^. Mussels are rich in protein, omega-3 fatty acids, and essential vitamins and minerals, making them a healthy food choice^10^.

Marine population connectivity plays a crucial role in distribution, recruitment, and stability of marine species, affecting the fishery industry and aquatic wildlife^11^. The lifecycle of many marine organisms incorporates a pelagic larval stage with sessile adults, where connectivity only occurs via dispersal of larvae in the planktonic stages. The pelagic stage is relevant to understanding of population dispersal patterns, as well as the timing and magnitude of recruitment^12^. Mussels have a complex life history, making it important to understand their dispersal and settlement patterns to manage and conserve both wild and farmed populations. Nevertheless, barriers to gene flow in the marine pelagic environment are often not clearly identified^13^ and must be studied in relation to hydrology and hydrography^14,15^.

Larval development of blue mussels begins shortly after fertilisation, and consists of two motile stages, the non-feeding trochophore and feeding veliger, then one partially motile stage, the pediveliger. The whole pelagic larval stage lasts three to four weeks, depending on temperature and food availability, during which time the principal organs (foot, digestive gland, and gills) begin to develop^16,17^. After three to four weeks, veliger larvae are fully developed pediveligers, ready to settle and metamorphose: an irreversible step in the bivalve lifecycle. As the pelagic larval stage alone is capable of greater dispersal, the fate of larvae is a key determinant of population connectivity^11,18^.

Management of bivalve populations for sustainable food production goes hand in hand with adequate conservation of spawning populations in the wild. In the case of mussels, this means gaining a clear understanding of how larval connectivity, habitat locations and environmental conditions allow the population to maintain a stable, connected network. In turn, this requires an understanding of both the genetic structure of populations and the biological and physical processes that generate this^19,20^.

Biophysical models are a useful tool to study larvae dispersal and connectivity in marine environments. These models replicate both the physical and biological mechanisms that shape larval transport within the ocean, encompassing vital factors such as ocean currents, turbulence, and larval behaviour^21^. Comprising three fundamental constituents, such models consist of a hydrodynamic module for the emulation of ocean currents and transport phenomena, a particle-tracking module that reproduces larval movement and responsiveness to environmental cues, and a biological component designed to replicate processes such as growth, mortality, and settlement^22,23^. Furthermore, biophysical models can provide insights into the processes driving population structure. However, in isolation, they do not describe the realised connectivity, and require verification by empirical methods. Depending on the species of interest, a range of approaches are available for verification, from otolith microchemistry^24^ to a wide range of genetic methods. Genetic methods are a powerful tool for understanding population structure, particularly in species with high levels of genetic diversity and complex life histories^25^. These methods can help researchers identify distinct populations, estimate gene flow between them, and assess the degree of genetic diversity within populations. Population genetic studies have advanced dramatically over the last twenty years, enabling much more refined distinction between local populations, e.g., sea lice population genetic studies^26,27^. Additionally, population genetic studies can combined with other approaches such as biophysical models to gain more comprehensive understanding of population structure and connectivity in marine environments^28^. For example, genetic methods can be used to validate biophysical models predictions of connectivity between populations or to identify sources of recruits to populations that are experiencing declines^29^.

In this study, we employed a multidisciplinary approach to investigate patterns of population connectivity and larval dispersion of blue mussels (*Mytilus edulis*) in Scottish waters. Genetic data enabled us to estimate population divergence, genetic connection and infer gene-flow patterns, while particle modelling allowed prediction of larval migration throughout the pelagic phase. Combining these two methods generated a comprehensive understanding of the population dynamics of blue mussels in Scottish waters and the factors influencing dispersal and population connectivity. The results of this research contribute to the conservation and management of this economically important species by providing insights into its population structure and dynamics.

## Results

### Spatial structure of mussel populations

**Fig. 1:**
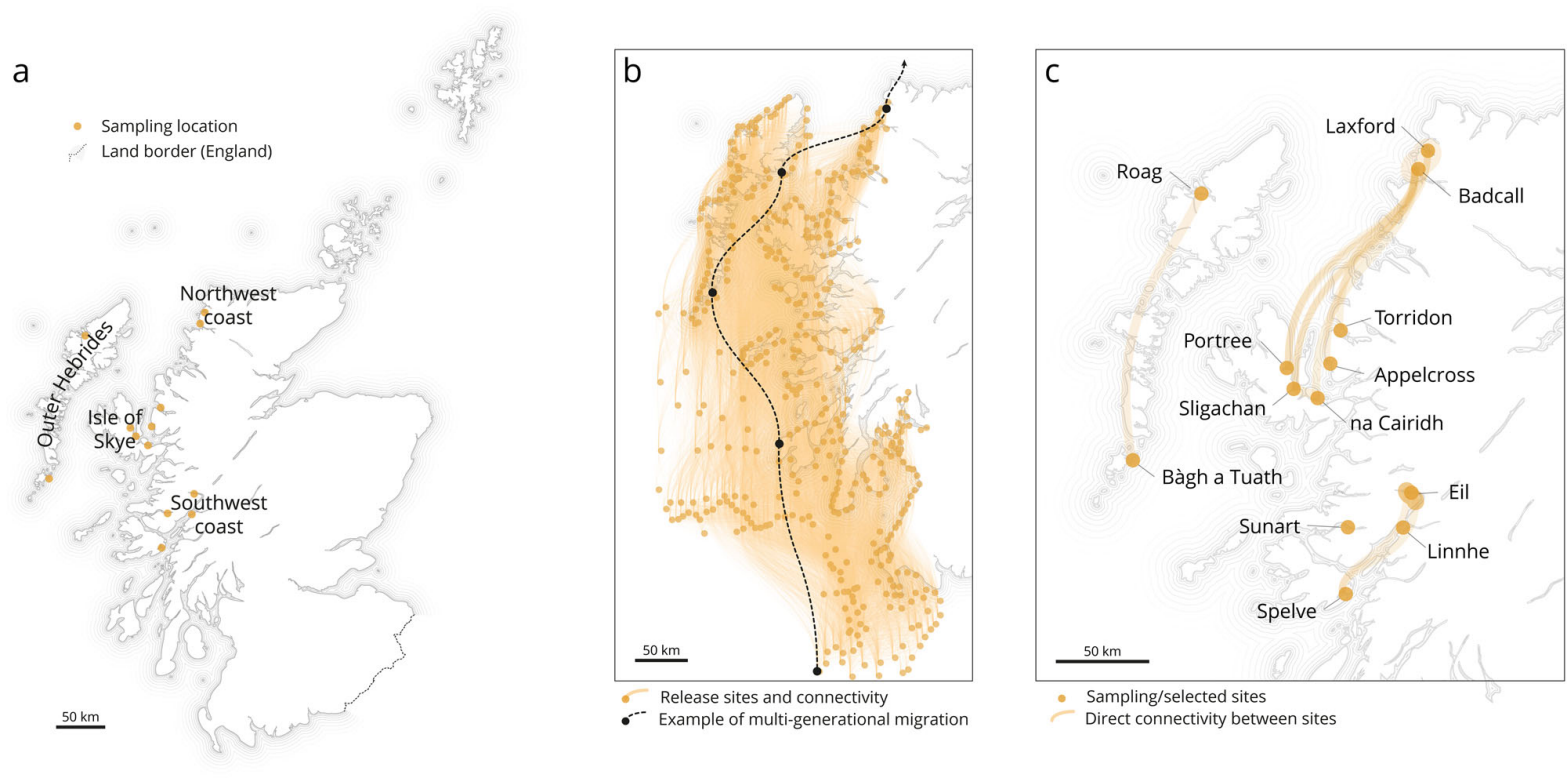
Hydrodynamic modelling and particle tracking. **a** General study location (west coast of Scotland); sampling sites are located in orange. **b** Connectivity network between all the 440 sites, in 2021 (light orange tracks). All south-west sites are along of Northern-Irish coastline (not depicted for simplicity). An example of multi-generational larval dispersal is illustrated by the black dotted line, a black circle identifies each settlement site. **c** Connectivity network between sampling sites only over the same period (simulated period, April 5th to May 8th, 2021).

Particle modelling using the WeStCOMS v2 hydrodynamic model indicated a predominant directional transport of particles from south to north, following major coastal currents along the mainland^30^. This was observed through the release of particles at 440 sites along the north coast of Ireland and west coast of Scotland (including both sites with mussel farming activities and wild mussel populations, plus randomly selected sites located homogenously along the west coast); There are strong connections northward between sites (Fig. 1b, c, and Supplementary Data 1).

This movement of particles is consistent with the estimated patterns of larval dispersal across multiple generations^30^. The particles, which originate from the southern limit of the model/region, move northward before settling and require several spring–neap tidal cycles to reach the northern limit of the model, with an average of three dispersal events between “stepping stones” being necessary. Figure 1b (black dotted line) illustrates an example where five such dispersal events were required. The extensive network of connectivity between sites is a result of the strong currents and surface winds in the region, creating a rapidly moving environment (Fig. 1b). The connectivity in Fig. 1c (and Supplementary Data 1) shows Lochs Eil, Linnhe, and Sunart as self-recruitment locations; Eil and Linnhe—located in proximity to each other—receive particles from the same source locations: Eil and S196 (source location near Eil). Sunart’s only source of particles is the source location in Sunart. The rest of the sampling locations receive particles from more than five other source locations.

In contrast, short-term connectivity (single year/“stepping stone”) is extensive (large number of particles) but limited in distance, with only sites in the vicinity connected (Fig. 1c). In fact, each site receives a large number of particles from multiple sources and distributes particles to various locations in turn (Fig. 1b). There are a few notable examples of this trend: the Outer Hebrides, where Bàgh a Tuath (off the Isle of Barra) only connects to Loch Roag; the sites off the Isle of Skye export particles only to sites on the northern west coast; and the tight cluster of sites on the southern west coast. Additionally, Loch Spelve, Loch Eil and Loch Linnhe are net donors, with Loch Eil being particularly connected to numerous sites despite its inland location (receiving almost no particles but being a significant source of particles).

### Genetic distances and population structure

We collected 520 blue mussels from 13 locations along the west coast of Scotland (Table 1) and used ddRAD sequencing to genotype them (EBI Project accession PRJEB52177). A total of 970,858,642 raw paired-end reads were sequences. After cleaning, 713,556,146 (73.5%) were mapped to the *M. edulis* genome. We identified 284,984 distinct RAD-loci and narrowed them down to 552 informative SNP markers for further analysis (Supplementary Data 2).

**Table 1:** Blue mussel sampling information.

Sampling site	Date	Latitude	Longitude	Stages	Remarks
Loch Eil	Oct 2020	5.21933°W	56.85365°N	Adults & Spats	Farm site
Loch Linnhe	Mar 2021	5.14962°W	56.80510°N	Adults & Spats	Farm site
Bàgh a Tuath	May 2022	7.38155°W	56.99230°N	Adults	Oyster farm facilities
Portree Bay	May 2022	6.14700°W	57.42000°N	Adults	Coastal sampling
Applecross Bay	May 2022	5.84500°W	57.44000°N	Adults	Coastal sampling
Loch Torridon	May 2022	5.74300°W	57.60000°N	Adults	Coastal sampling
Loch Sunart	Oct 2020	5.62250°W	56.67897°N	Adults & Spats	Farmed from Loch Eil
Loch Spelve	Sep 2021	5.72428°W	56.40262°N	Adults & Spats	Farm site
Loch Roag	Jun 2021	6.84620°W	58.20171°N	Adults & Spats	Farm site
Badcall Bay	Mar 2021	5.15477°W	58.31498°N	Adults & Spats	Decommissioned fish farm
Loch Laxford	Mar 2021	5.05577°W	58.39468°N	Adults & Spats	Farm site
Loch na Cairidh	Sep 2021	5.93147°W	57.27781°N	Spats	Coastal sampling
Bo Sligachan	May 2022	6.09293°W	57.32606°N	Adults	Coastal sampling

A total of 520 blue mussels were collected across the 13 sites.

**Fig. 2:**
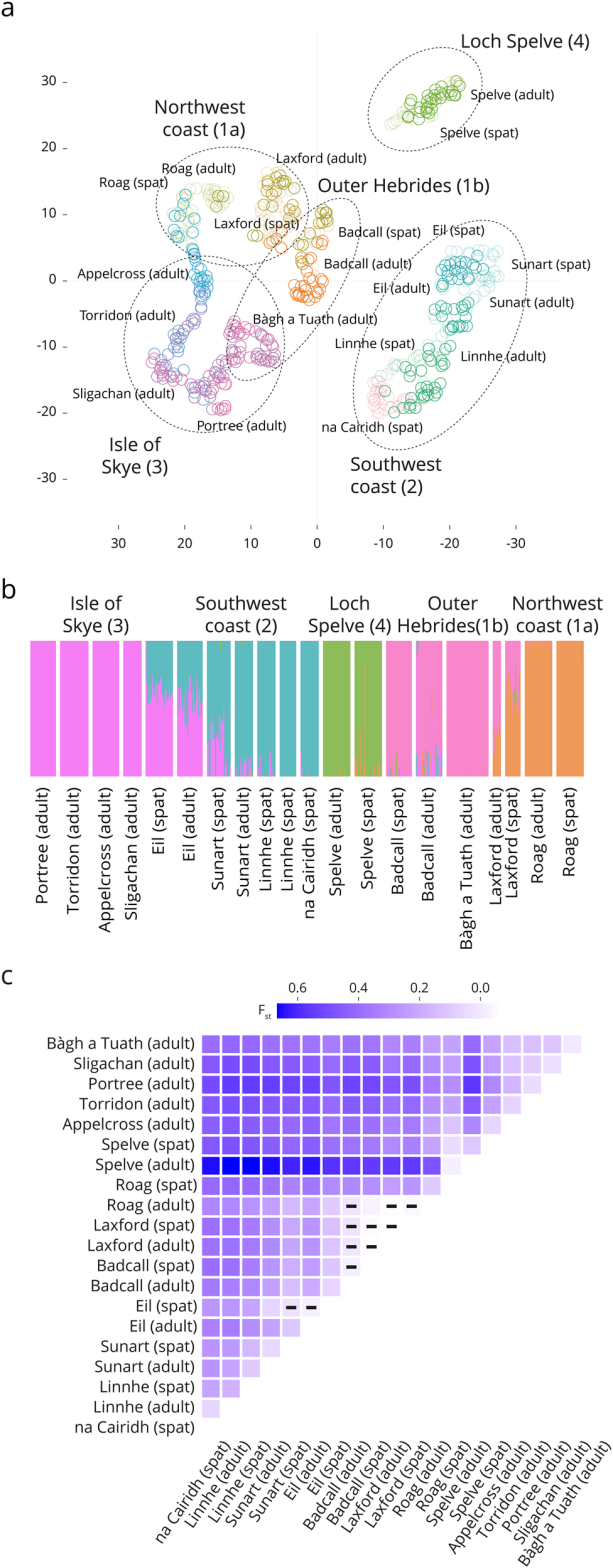
Blue mussel genetic analysis. **a** t-SNE. Individuals are broadly grouped geographically: Northern part of the west coast, Southern part of the west coast, the Outer Hebrides, The Isle of Skye area, and Loch Spelve (each sampling sites, Table 1, is presented with a different colour, spat of each site are in a light shade). **b** Genetic clustering analysis, each colour represents a different genetic cluster (K = 5 populations, one colour per population/group) of potential populations. **c** Genetic distance between each population and time points. The F_st_ values are reported by the colour shades. Non-significant distances are reported by a black dash (-).

A t-SNE analysis (Fig. 2a) revealed the presence of five genetic clusters, each of which corresponded broadly to a geographic region: (1) the northwest coast/Outer Hebrides, (2) the southwest part of the coast, (3) the Isle of Skye, and (4) Loch Spelve. This population structure was further supported by membership assignment probability (Fig. 2b), which suggested gene flow between these regions and clarify the complex northern part of the west coast by slitting it in two broad clusters: (1a) the north part of the west coast (Loch Roag and Loch Laxford) and (1b) the central part of the west coast (and Badcall Bay). The maximum likelihood model for this data (with K = 5 populations) separated the southern individuals into a unique cluster, specifically isolating the samples from Loch Spelve.

The genetic distances (F_st_ values), acting as genetic differentiation indices, were calculated to further explore the relationships between populations. F_st_ values calculated on the 552 informative markers (Fig. 2c) between all pairs of populations were statistically significant, except for three higher-latitude locations (Loch Roag in the Outer Hebrides, Badcall Bay, and Loch Laxford on the northwest coast) and two locations in the southern west coast (Loch Eil and Loch Sunart). Notably, the highest F_st_ values were observed in adult samples collected from Loch Spelve when compared to the other locations: Loch Spelve appears to be genetically more isolated. These findings suggest a strong and ongoing exchange of genetic material between populations from year to year, shaping the evolution of these populations from one year to another. It is worth noting that when considering all 284,984 loci, the F_st_ value is substantially lower at 0.01.

### Model comparison with genetic data

**Fig. 3:**
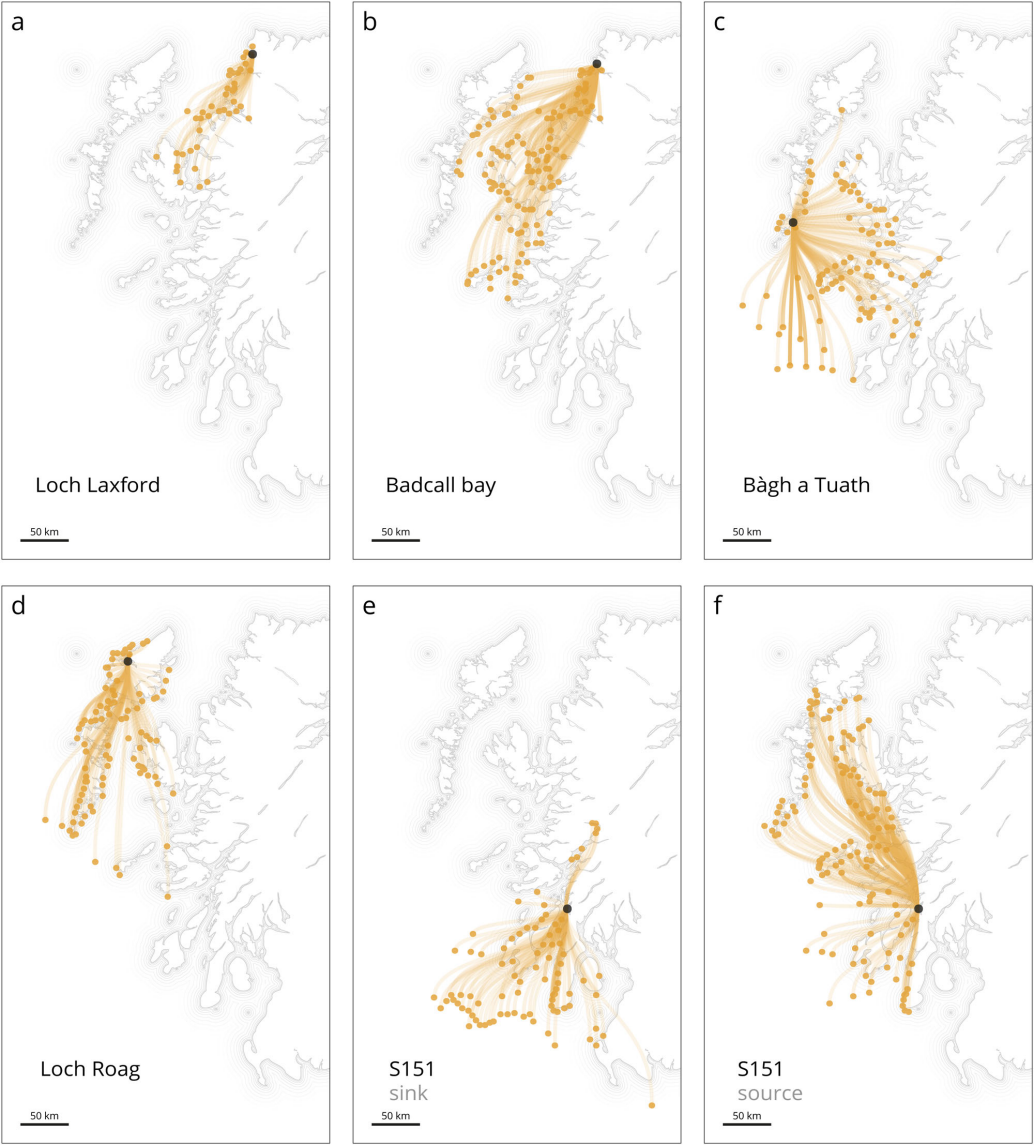
Example of locations acting as source for the several mussel locations of interest. **a**–**f** Locations acting as source (light orange tracks) for the several mussel locations of interest (black dot). In total 229 locations out of 440 are sending samples to the mussel farms of interest. S151, acting as a sink (**e**) and source (**f**) for samples in higher latitudes.

From the biophysical model, we located the source of the mussel populations at the 13 mussel-sampling sites along the west coast of Scotland. By examining the connectivity matrix used for Fig. 1b (Supplementary Data 1), we were able to identify a total of 229 locations out of 440 as the sources for these mussel populations (April 5th to May 8th, 2021; Fig. 3). The results align with our genetic findings, revealing a strong connection between the upper southern coast and the rest of the west coast. Mussel locations such as Badcall Bay, Bàgh a Tuath, Loch Roag and Loch Laxford receive larvae from 119, 98, 93, and 42 different source locations, respectively (Fig. 3a–d).

To further understand the dispersion patterns of these mussels, from the 229 locations previously identified, we selected a “stepping stone” source location in the southern west coast. As shown in Fig. 3e, the source location S151 (southwest coast) receives larvae from 87 different locations and in turn, disperse larvae to 127 other locations (Fig. 3f), including the location of Bàgh a Tuath (Outer Hebrides). This behaviour is further supported by our population structure and genetic results, which identifies southern mussel farms as a major primary source for the rest of the locations. Specifically, we observe that the mussel population at Spelve receives particles primarily from its own location and just nine other source locations, with Loch Eil and Linnhe being the most substantial contributors. Our genetic results concur with this pattern, as Loch Spelve exhibits higher levels of genetic differentiation (Fig. 2c) and forms a unique cluster influenced almost uniquely by Loch Eil and Linnhe.

**Fig. 4:**
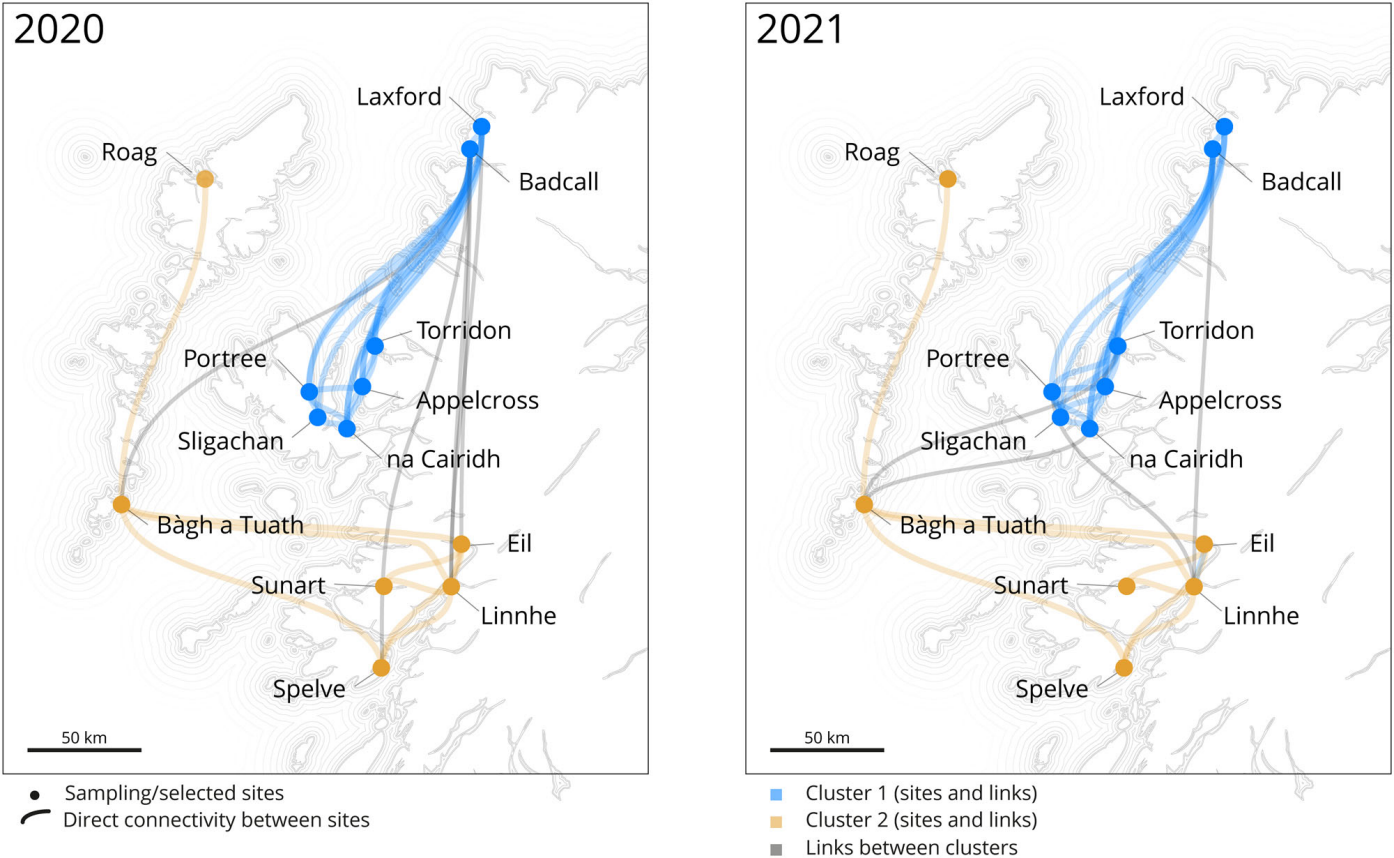
Cumulative networks for 2020 and 2021. Considering the asynchronous nature of the spawning events occurring across a range of locations, we conducted simulations for 34-days starting every week between February and June of both 2020 and 2021. The cumulative results are reported. The clusters (coloured nodes and links) are the densely connected sub-graphs via random walks. Details week per weeks are available in Supplementary Figs. 1 and 2.

Accounting for the asynchronous nature of the spawning events occurring across a range of locations, we conducted simulations for 34 days, starting every week between February and June of both 2020 and 2021 (Fig. 4 and Supplementary Figs. 1 and 2). This allowed us to explore the possible connection between the population genetic structure and the particle migration. Our analysis revealed sampling locations grouped in two main clusters (southwest coast with Outer Hebrides, and Isle of Skye with northwest coast). Simulations for both 2020 and 2021 demonstrated similar temporal trajectories for the particles, indicating a connection between genetic population structure and particle movement.

There is one notable disagreement between the genetic data and the WeStCOMS v2 hydrodynamic model transport: the Bo Sligachan location. The genetic data indicates a strong connection with the southern west coast cluster, whereas the model does not show a direct link between the two locations, restricted by very shallow region under the Skye Bridge with periodic exposure of seabed under low tides.

### Model predictions

**Fig. 5:**
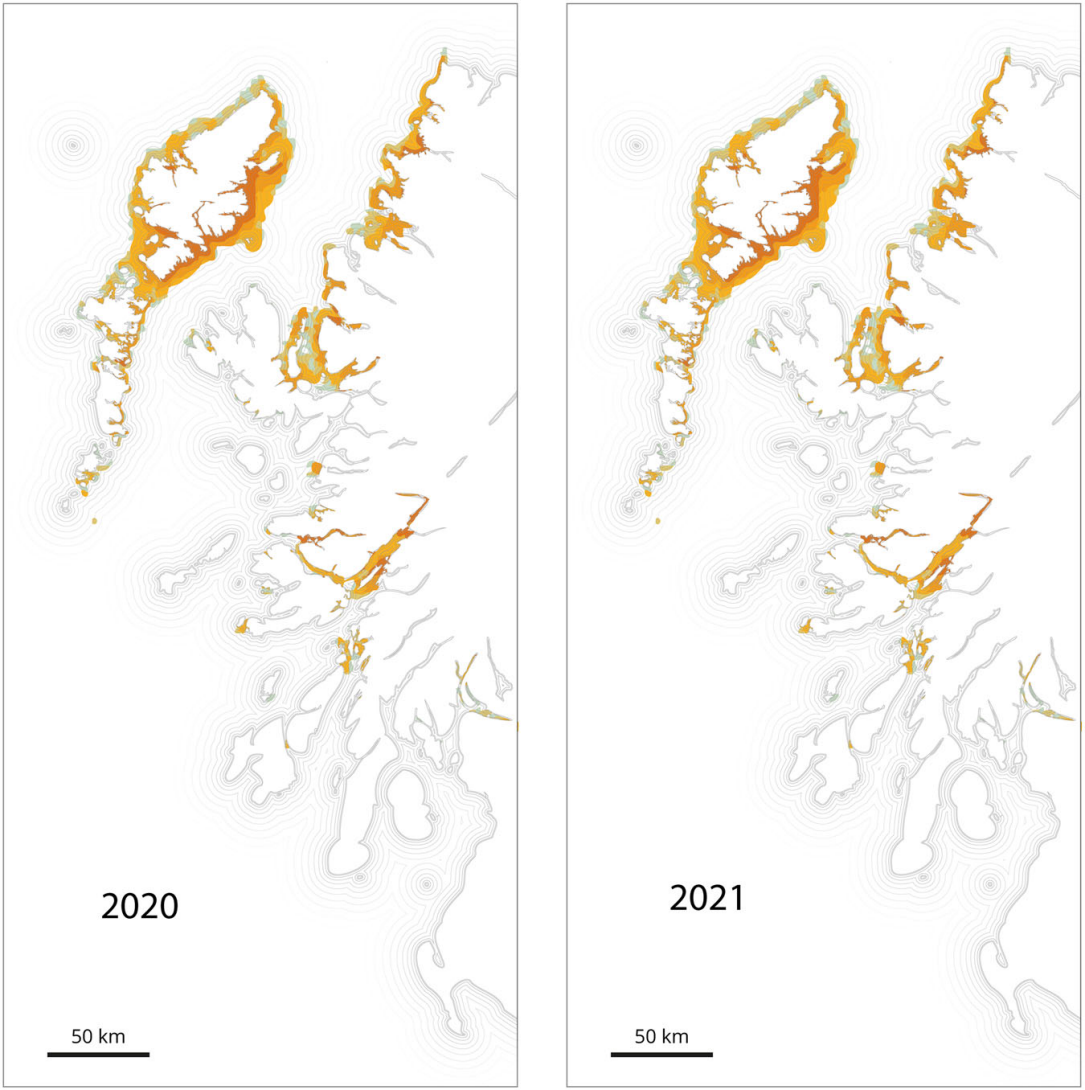
Predicted areas of accumulation of blue mussel larvae, based on 2020 and 2021 simulation and hydrodynamic data.

The particle accumulation of larvae (particles/m^3^) predicted by the model (Fig. 5) predicts those areas with the likely highest density of blue mussel larvae for both 2020 and 2021, assuming that all modelled sites have equal larval output. Simulations of the 440 release sites (Fig. 1b), highlights three main areas of particle accumulation: the northern section of the Outer Hebrides; the northern mainland (from Skye to the northern limit of the model); and the southern west coast area, specifically near Loch Eil.

Although Fig. 5 predicts higher accumulation in these three areas, genetic analysis also reveals that locations on the north and central west coast are genetically more diverse and influenced by other sampling locations, and, to a lesser extent, the locations in the Isle of Skye area (Fig. 2). The East side of the Outer Hebrides shows a particularly high accumulation of particles, however, validation here is lacking since we do not have genetic information from this area. Based on the consistency between our simulation and genetic data, it is possible that the eastern part of the Outer Hebrides could be a genetically diverse area for mussel farming.

## Discussion

Our study represents the first integrated study in Scotland that combines population genomics and particle modelling to enhance spatial and temporal understanding of population connectivity for the commercially significant mussel species *M. edulis*. By combining it with our prior research^30^, this approach demonstrates the genetic exchange that occurs among populations, even those separated by great distances and impacted by oceanographic factors. To date, there have been few studies that have combined population genomics and particle modelling in *M. edulis*. Studies such as refs. ^29,31,32^, have explored *M. edulis* connectivity in the Limfjorden, North Sea, and Baltic Sea, respectively. On the other hand, some studies have focused solely on hydrodynamic larval particle tracking models, such as refs. ^33–35^, which investigate *M. edulis* connectivity in Belgium, Ireland, and Maine, respectively. Additionally, some studies have exclusively examined genetic diversity, like refs. ^36–38^, to explore *M. edulis* connectivity in Wales, the Arctic, and Maine, respectively. Research on population connectivity of *M. edulis* in Scotland is relatively limited, and we argue that the most effective approach to studying this connectivity involves combining hydrodynamic larval particle-tracking models with in-situ spat and adult sequencing. This method provides a more complete understanding of the genetic structure of mussel populations and their dispersal patterns, ultimately allowing for more accurate predictions of future population dynamics.

Overall, the west coast of Scotland shows a strong physical link between southern and northern areas maintained by the regional water circulation. Looking at particle dispersal only, the thirteen areas of interest (sampling locations) appear connected with the closer sampling sites only (Fig. 1c). However, the thirteen areas of interest received particles from multiple other locations (Fig. 3). Looking at the genetic structure only, we found five genetic clusters grouped by geographical regions: going from southern areas to the northern part of the west coast (southwest coast, Loch Spelve, Isle of Skye, Outer Hebrides, and northwest coast). The rapid water movement and larval transportation in Scottish waters result in a genetically diverse population of mussels. If genetic analysis were to be conducted again on new collected samples, the resulting clusters would likely be similar, although the relative positioning of each population would differ. This is due to a continual sharing and shifting of genes, which becomes more apparent when comparing successive generations (i.e., spats and adults) of the mussel populations (Fig. 2a). This genetic mixing is due to the high levels of connectivity facilitated by water currents, which allow for the exchange of genetic material between different mussel populations. Therefore, it can be inferred that the genetic diversity of mussels in Scotland is constantly evolving, with new genetic clusters forming as populations mix and exchange genes. The disagreement between the genetic data and the WeStCOMS v2 hydrodynamic model transport for the Bo Sligachan location could be explained either by the presence of an indirect connection that is not easily captured by the genetic data, or by the inaccuracy of the simulating for the unique conditions of the Bo Sligachan location. Another possibility is that the northern entrance to the Sound of Raasay is sufficiently wide to allow aperiodic influx of the waters from the Minch Sound between Skye and Outer Hebrides, occasionally delivering here the particles (larvae) from the southwestern coast cluster.

The connectivity among mussel populations on the west coast of Scotland suggests that some areas act as net sources or sinks. Specifically, our analysis indicates that the mussel farm in Loch Eil, which belongs to the southern west coast cluster, serves as a source of larvae, while areas in the Outer Hebrides and northern west coast, such as the mussel farms in Loch Roag and Badcall Bay, act as sinks, receiving larvae from other locations. The extent of larval supply to these sink stocks is likely influenced by the hydrodynamic regime and the intermediate blue mussel population involved in the transfer process. Despite potential genetic differences, this exchange seems to be sufficient to support connectivity among the areas and create a well-mixed genetic pool. Sink stocks, like those found in Loch Roag and Badcall Bay, play an important role in maintaining genetic diversity within a population. This diversity can provide benefits such as increased resilience to environmental stresses, improved adaptability to changing conditions, and greater fitness, ultimately promoting the long-term survival of the population^39^. However, the survival of sink stocks depends on the availability of larvae from source stocks with sufficiently large effective population sizes and self-recruitment rates to maintain themselves, such as those found in Loch Eil and S151 (Fig. 5). Overexploitation of source stocks, pollution, and climate change can have negative impacts on the viability of the entire population, including decreased recruitment, productivity, and persistence of associated sink stocks^15^. Therefore, the importance of maintaining source stocks with large effective population sizes and high self-recruitment rates cannot be overstated.

This highlights the importance of studying population connectivity to better understand the genetic structure of mussel populations and how they may be impacted by factors affecting the accuracy of genetic analyses and the conclusions drawn about mussel connectivity^18,29,40,41^. We cannot completely exclude the possibility of spat import from other areas in samples collected from the farms, and there might be a different genetic composition between natural mussel beds and rope cultures due to settlement and post-settlement selection^42^, especially in the areas where hybridisation between *M. edulis*, *M. galloprovincialis*, and *M. trossulus* has been reported, such as the Loch Eil region in 2010^43,44^. The concern arises because hybridisation can introduce new genetic characteristics into the population, which may disrupt local adaptation and genetic integrity. This can be problematic for understanding population connectivity and the genetic structure of mussel populations, especially when studying how factors like larval transport or water re-circulation patterns impact genetic exchange between different locations. In this study, due to transfer from the same farmer’s mussel farming operations, samples collected from Loch Eil and Loch Sunart exhibited identical genetic patterns. However, the biophysical modelling results did not show a direct connectivity between Loch Eil and Loch Sunart (Fig. 1c), indicating that other factors such as larval transport or water re-circulation patterns (i.e., around the Isle of Mull) are capable only occasionally or even may not facilitate significantly genetic exchange between these two locations on a local scale.

The west coast of Scotland is a well-connected system for mussel dispersal and recruitment. Identifying the key source and sink areas for mussel larvae is important for decision-making related to mussel farming, and crucial for the ecosystem as a whole^45^. For example, Loch Eil, in the southern west coast has been identified as a key source area for mussel larvae; it may be important to protect this area to ensure a sustainable supply of mussel larvae for other areas. Similarly, Loch Roag and Badcall Bay have been identified as key sink areas; it may be important to focus mussel farming efforts in this area to ensure a healthy and sustainable population of mussels. Mussels play an essential role in supporting other marine organisms and maintaining water quality: By ensuring sustainable populations of mussels through responsible farming practices and protecting key source areas, we can contribute to the overall health and resilience of marine ecosystems in Scotland.

Recently, the Marine Scotland Science Scottish Shellfish Farm Production Survey 2021 has reported the presence of the parasitic *Bonamia ostreae*, mainly in the southern west coast region and more specifically in Loch Sunart, and movement restrictions have been imposed in both these areas. In the past, Scottish farmers have also reported fluctuations in spat recruitment and settlement^2,3,46^, which supports the idea that preventing mussel production in important source areas could reduce the supply of mussel larvae to other regions, affecting the overall productivity and profitability of the mussel farming industry in Scotland. Therefore, it is crucial to carefully consider the potential consequences of halting production in areas where the source of larvae is abundant and supplies multiple other regions. Any decisions related to the management and protection of these areas should be based on a thorough understanding of the ecological and economic factors involved, balancing the needs of the industry with the need to protect and maintain the health of the broader marine ecosystem.

There are alternatives to completely restricting production in key source areas, such as mussel farms in Loch Eil. Simple and effective ways for farmers to maintain the genetic integrity of mussel populations include establishing buffer zones^47^ around the farming area where no mussels are cultivated. This reduces the chances of hybridisation occurring between farmed and wild populations and ensures a sustainable supply of mussel larvae for other areas. Additionally, buffer zones can help to protect the long-term health and productivity of the broader marine ecosystem^48^. The monitoring and controlling mussel populations, the regular tracking and removal of individuals showing signs of hybridisation, and the use of regular genetic testing of mussels are other options^49,50^. In northern areas where mussel farms receive seeds from multiple source locations, reports of hybridisation events or disease spread are infrequent. Farmers can obtain mussel seeds from multiple sources, to reduce the risk of introducing hybrids into their farming area, maintaining genetic diversity, and improving the overall health and resilience of the local mussel population.

Consideration of management alternatives is important for mussel farming and stock recruitment, and identification of key source and sink areas for mussel larvae is crucial for decision-making for mussel farming in Scotland. Connectivity patterns are important ecological features for stock recruitment by indicating which local stocks rely on larval retention and self-recruitment and versus migration^51^. Changes in mussel connectivity, whether due to natural variability or as a result of human activities, can have significant impacts on the dispersal and recruitment of mussel larvae. Thus, understanding mussel recruitment processes can also help to address climate change concerns in specific areas. By implementing strategies such as hatchery production monitoring, and controlling mussel populations, establishing buffer zones, conducting regular genetic testing, and diversifying seed sources, farmers can help to maintain the genetic purity of local mussel populations, reduce the risk of hybridisation, and contribute to the overall health and resilience of marine ecosystems in Scotland.

In this research, the initial hypothesis underlying the study posited that each target location would have one or two source locations, and the sampling strategy was designed accordingly, i.e., sampling adult and spat mussels from nearby areas in each mussel farm. However, a more nuanced and intricate picture emerged from the findings, which were further refined by the advanced biophysical model. The results suggesting that each site consists of a complex assemblage of self-recruitment and a vast network of sources, which creates a mesh-like pattern that cannot be readily tested through conventional genetic means. Indeed, meaningful validation of the fine details would require a rigorous and protracted sampling effort, involving weekly collections from each of the 440 source locations. And even though we represented a simulation of particles (bivalve larvae) moving along the West coast of Scotland, our model has significant limitations that will need to be addressed in future studies. For example, the particle-tracking model does not account for vertical migration of particles through the different layers of the FVCOM mesh, and for biological parameters such as temperature and salinity as little data are available to model these phenomena and no mortality was considered as data are lacking concerning mortality rates for larval bivalves. Finally, the ddRAD analysis did not yield the expected number of markers, which can be attributed to the genome-duplication events in *Mytilus* evolution^52^ or to inadequate ddRAD library preparation and sequencing issues^53^. Alternatively, there are several factors that can contribute to obtaining a low number of markers during ddRAD library preparation, including DNA quality and quantity, library preparation protocol, and PCR amplification bias, which can impact the number of markers generated^54^.

For future improved studies, mapping mussel populations to areas beyond the west coast of Scotland could give a more complete understanding of larger-scale patterns of mussel dispersal. Furthermore, to assess migration rates efficiently, it is recommended to collect the samples from the same year/season to minimise any potential biases due to temporal variability. In addition to monitoring the changes in mussel populations, investigating the effects of climate change on mussel connectivity is also crucial: Climate change is expected to disrupt larval dispersal through changes in temperature, salinity levels, and current patterns, potentially leading to declines in mussel populations and shifts in their distribution^55–57^. Understanding how these changes may impact mussel populations is important for developing effective management strategies to maintain their health and sustainability. Overall, continued research into mussel connectivity and the factors that influence it is essential for the long-term conservation of these valuable resources. By better understanding how ocean and coastal circulation patterns affect mussel populations and developing strategies to adapt to changes, we can ensure the continued productivity and resilience of marine ecosystems in Scotland and beyond.

Our study emphasises the importance of understanding the genetic dynamics and connectivity patterns of mussel populations in Scotland. We find a continuous mixing of genetic material between locations, leading to rapid evolution and changes in local populations. This highlights the need for effective management strategies to maintain genetic integrity, reduce the risk of hybridisation, and ensure a sustainable supply of mussel larvae for other areas. These findings can inform decision-making for mussel farming and conservation efforts in Scotland. Future work should focus on developing and implementing management strategies that account for the genetic connectivity patterns of mussel populations and concomitant ecological impacts.

## Methods

### Institutional review

Animal handling and collection in this study was carried out in accordance with the UK Animals (Scientific Procedures) Act 1986 Amended Regulations (SI 2012/3039) and the work was approved by the University of Stirling Ethics Committee (Animal Welfare and Ethics Review Board).

### Study samples

The study was undertaken on the western coast of Scotland, where the primary mussel producers strategically position themselves in dynamic shoreline regions. We collected a total of 520 blue mussels from 13 different locations along this coastal stretch, averaging approximately 40 samples per site. These samples were obtained from natural mussel beds located along the coast in Portree, Applecross Bay, Loch Torridon, Loch na Cairidih, and Bo Sligachan, as well as from mussel farms in Loch Eil, Loch Linnhe, Bàgh a Tuath, Loch Sunart, Loch Spelve, Loch Roag, Loch Laxford, and Badcall Bay (Fig. 1a and Table 1). We then took tissue samples, including gills, from the adult mussels and all body tissues from the spats and stored them in 99% ethanol at a temperature of −20 °C.

### Particle modelling

The particle-tracking method developed in our previous study^30^ was applied. It used 3D current fields, derived from the Finite Volume Coastal Ocean Model (FVCOM) hydrodynamic model^58^. The latest regional implementation of coupled atmospheric and ocean WeStCOMS v2 model system became operational in April 2019 for producing flow hindcast and forecast^59,60^. Particle tracking was conducted using the BioTracker model^22,23^. This predicts the dispersal of particles due to the interaction of physical processes such as tidally and wind-driven water movements, with biological processes such as maturation and mortality. In this study, maturation and mortality were omitted from the particle definition, but instead a settlement window for calculation of successful dispersal was applied during simulations. The particle-tracking code can be found at https://github.com/tomadams1982/BioTracker (commit 9fbf1bb).

For the simulations, particles were released from 440 randomly selected distinct locations, and from the specific sampling sites from where mussels were collected, at 6 metres below the sea level: Our previous research study^30^ showed no significant differences when releasing the particles from three different depths. This approach was chosen to ensure a comprehensive and unbiased representation of particle dispersal patterns in the region. By employing random release sites, we aimed to minimise potential biases that could arise from selecting specific locations based on preconceived notions or prior assumptions. The 440 selected locations ranged from the Isle of Man in the south to Cape Wrath in the north, and westward to the Outer Hebrides archipelago. Twenty simulated particles were released per hour at each location. Particle tracking simulations lasted 34 days, for which particles were released over the first 14 days every hour, and accumulations were counted for the last 7 days within a 2 km radius of each sampling point to estimate settlement; particles did not stop moving, allowing us the use of a 7-day window to account for settlement/accumulation of particles. To account for the asynchronous aspect of the spawning between locations, simulations were run every week, between 2020-02-01 to 2020-05-02 and 2021-02-01 to 2021-05-03 (years of the larval dispersal of the samples) using hydrodynamic, and wind fields collected for the same period. Connectivity matrices^30,61^ obtained from the particle tracking model simulations were analysed to identify source and sink locations.

### Sample preparation

Using an adapted version of a previously published protocol^62^, the genotyping data of 520 individuals was obtained through double-digest restriction-site associated DNA (ddRAD) sequencing. Briefly, genomic DNA was extracted using a salt-extraction method. All samples were tested for *M. edulis* species identity against the ref. ^63^ SNP panel, before further processing. The DNA of each sample was digested with restriction enzymes *Sbf*I and *Sph*I, which recognise the sequence CCTGCA^GG and GCATG^C, respectively. After adding a unique index to each sample, 550–650 bp DNA fragments were amplified and sequenced on the Illumina NextSeq platform, resulting in 150-nt paired-end reads (Edinburgh Clinical Research Facility, UK).

### Genotyping and variant calling

Using fastp v0.20.1^64^, we filtered the raw reads for quality (QC ≥ 25), length (150 nt), the absence of primers/adaptors, and complexity (entropy over 15). We then used BWA v0.7.17^65^ to map the clean reads to the *M. edulis* reference genome (MEDL1; GCA_905397895.1^52^). The resulting SAM files were converted into BAM files and sorted using SAMtools v1.16.1^66^. We used *gstacks* from Stacks v2.62^67^ to identify SNPs, and called all bi-allelic SNPs that were common to at least 50% of the individuals using *populations* (Stacks v2.62). We filtered these SNPs using PLINK v2.00a3.7LM, keeping only those with a minor allele frequency over 0.05 and not deviating from the expected Hardy–Weinberg equilibrium (*P*-value of χ^2^test ≥ 10^−8^). The threshold for the *P*-value cut-off was determined empirically, by examining the spread of *P*-values from the Hardy–Weinberg test in the data and selecting a threshold under which there are a greater number of variants than expected by chance (in our experience, with small data sets, this is typically around *P* = 10^−8^). Finally, we used Beagle v5.4-22Jul22.46e^68^ with the GT parameter to infer missing data.

### Connectivity and population structure

Dimension reduction and visualisation were carried out using a t-distributed stochastic neighbour embedding (t-SNE) analysis^69^, as implemented in the Rtsne v0.16 package^70^. Additionally, we used FastSTRUCTURE v1.0^71^, with the logistic prior admixture model, to evaluate the population structure and admixture. The optimal number of populations, or K, was determined by maximising the marginal likelihood. To examine the amount of genetic differentiation among sampling localities and years, pairwise F_st_^72^ with 1000 bootstrap replicates and P-values were calculated between sites using the HIERFSTAT v0.5–11 package^73^. Community and network analysis were done using the function *cluster_walktrap*^74^ from the igraph v1.3.4 package^75^. Two different clustering methods were used to analyse mussel populations: the t-SNE clustering method (genetic data) and the community and network clustering method (particle tracking simulations). The comparison of the clusters generated by the two methods allowed for a deeper understanding of the relationships between the mussel populations.

### Statistics and reproducibility

All statistical analysis and visualisation were performed using R v4.2.2^76^, and map visualisation was achieved using the Database of Global Administrative Areas (GADM) v4.1.

### Reporting summary

Further information on research design is available in the Nature Portfolio Reporting Summary linked to this article.

### Supplementary information

Supplementary Information

Description of Additional Supplementary Data

Supplementary Data 1

Supplementary Data 2

Reporting Summary

## Data Availability

The raw sequencing reads of all libraries are available from EBI/ENA via the project PRJEB52177. WeStCOMS hindcast and forecast modelling data since 2013 are publicly accessible at https://thredds.sams.ac.uk/. Data used to generate all figures are available at https://github.com/pseudogene/Corrochano-Fraile_et_al_2023.

## Code availability

The particle-tracking code can be found at https://github.com/tomadams1982/BioTracker (commit 9fbf1bb). Scripts and workflows used at available at https://github.com/pseudogene/Corrochano-Fraile_et_al_2023.
