# Supplementary material for: Estimating blue mussel (Mytilus edulis) connectivity and settlement capacity in mid-latitude fjord regions
Source: Commun Biol. 2024 Feb 9;7:166. doi: 10.1038/s42003-023-05498-3 (PMC10858254; doi:10.1038/s42003-023-05498-3)
Supplement: Supplementary file 1 — Supplementary Information [file 42003_2023_5498_MOESM1_ESM.pdf]

Estimating blue mussel (*Mytilus edulis*) connectivity and settlement capacity in mid-latitude fjord regions

Ana Corrochano-Fraile[1], Stefano Carboni[1,2], Darren M. Green[1], John B. Taggart[1], Thomas P. Adams[3], Dmitry Aleynik[4], and Michaël Bekaert[1,*]

[1]Institute of Aquaculture, University of Stirling, Stirling, United Kingdom
[2]Fondazione IMC, Torre Grande, Oristano, Italy
[3]Scottish Sea Farms Limited, Barcaldine Hatchery, Argyll, United Kingdom
[4]Scottish Association for Marine Science, Oban, United Kingdom
[*]corresponding author: Michaël Bekaert (michael.bekaert@stir.ac.uk)

Supplementary Figures

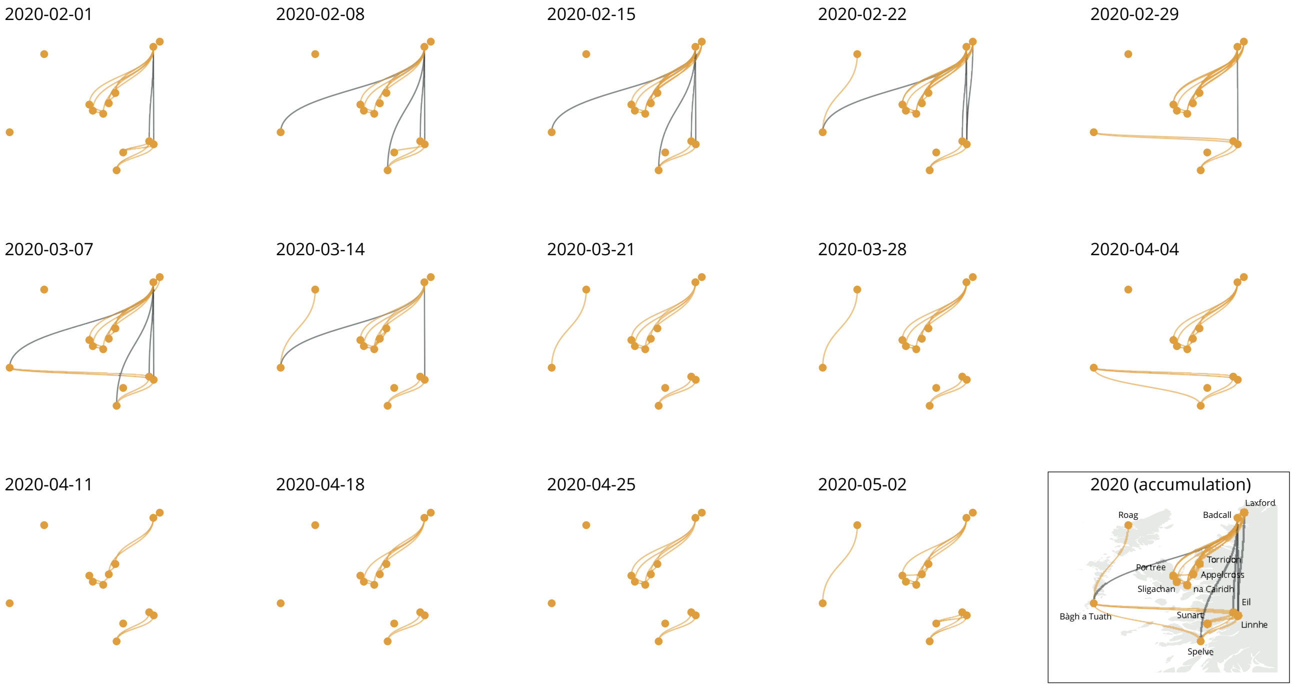

Supplementary Fig. 1. Simulations spanning 34 days from February to May 2020. Each panel illustrates the connectivity network, showing where the spats gathered in the final 7 days of the simulation and their origin (tracking lines). The start date of each simulation is reported.

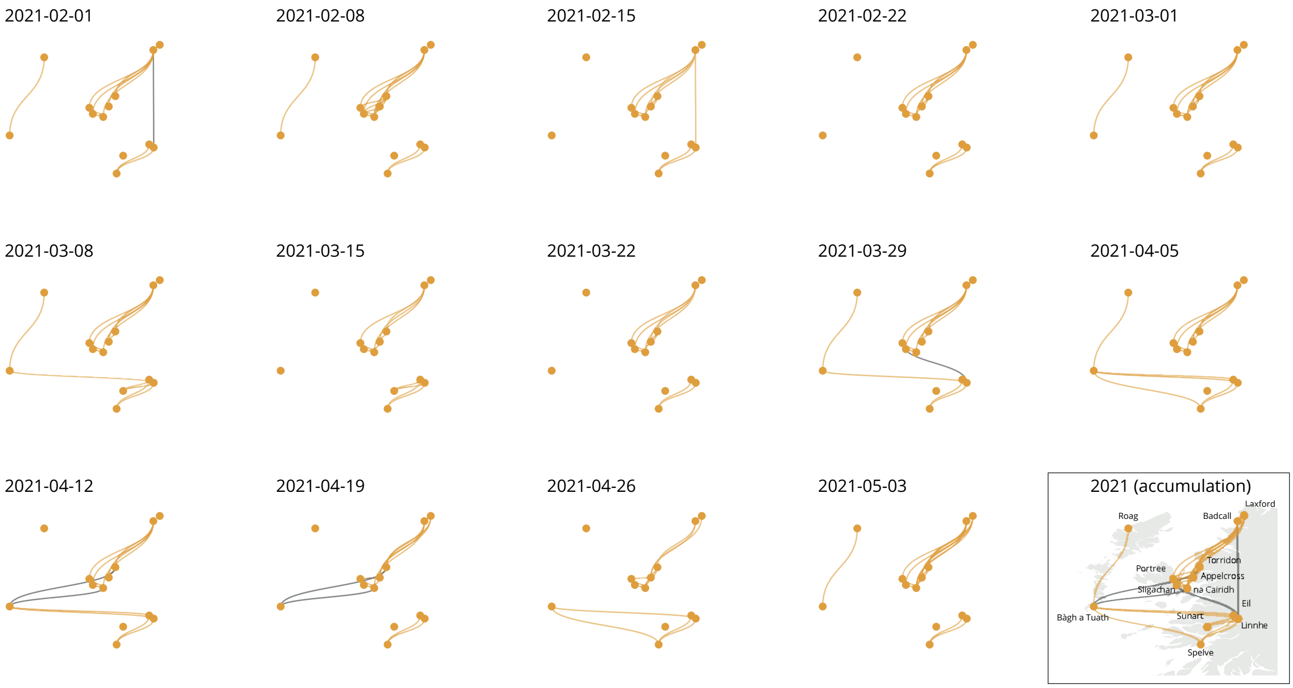

Supplementary Fig. 2. Simulations spanning 34 days from February to May 2021. Each panel illustrates the connectivity network, showing where the spats gathered in the final 7 days of the simulation and their origin (tracking lines). The start date of each simulation is reported.
