# Supplementary material for: Estimating blue mussel (Mytilus edulis) connectivity and settlement capacity in mid-latitude fjord regions
Source: Commun Biol. 2024 Feb 9;7:166. doi: 10.1038/s42003-023-05498-3 (PMC10858254; doi:10.1038/s42003-023-05498-3)
Supplement: Supplementary file 2 — Description of Additional Supplementary Data [file 42003_2023_5498_MOESM2_ESM.docx]

**Description of Additional Supplementary Files**

**File name:** Supplementary Data 1

**Description:** Connectivity matrix between source and target location over a simulated period, April 5th to May 8th, 2021. (CSV)

**File name:** Supplementary Data 2

**Description:** Genotypes of the 520 samples and 552 markers. Each marker is located on the GCA_905397895.1 assembly. (VCF)
