# Supplementary material for: Estimating blue mussel (Mytilus edulis) connectivity and settlement capacity in mid-latitude fjord regions
Source: Commun Biol. 2024 Feb 9;7:166. doi: 10.1038/s42003-023-05498-3 (PMC10858254; doi:10.1038/s42003-023-05498-3)
Supplement: Supplementary file 5 — Reporting Summary [file 42003_2023_5498_MOESM5_ESM.pdf]

nature portfolio

Corresponding author(s): Dr Michaël Bekaert

Last updated by author(s): May 29, 2023

Reporting Summary

Nature Portfolio wishes to improve the reproducibility of the work that we publish. This form provides structure for consistency and transparency in reporting. For further information on Nature Portfolio policies, see our Editorial Policies and the Editorial Policy Checklist.

Statistics

For all statistical analyses, confirm that the following items are present in the figure legend, table legend, main text, or Methods section.

n/a	Confirmed	
☐	☒	The exact sample size (*n*) for each experimental group/condition, given as a discrete number and unit of measurement
☐	☒	A statement on whether measurements were taken from distinct samples or whether the same sample was measured repeatedly
☐	☒	The statistical test(s) used AND whether they are one- or two-sided<br>*Only common tests should be described solely by name; describe more complex techniques in the Methods section.*
☒	☐	A description of all covariates tested
☐	☒	A description of any assumptions or corrections, such as tests of normality and adjustment for multiple comparisons
☒	☐	A full description of the statistical parameters including central tendency (e.g. means) or other basic estimates (e.g. regression coefficient) AND variation (e.g. standard deviation) or associated estimates of uncertainty (e.g. confidence intervals)
☒	☐	For null hypothesis testing, the test statistic (e.g. *F*, *t*, *r*) with confidence intervals, effect sizes, degrees of freedom and *P* value noted<br>*Give P values as exact values whenever suitable.*
☒	☐	For Bayesian analysis, information on the choice of priors and Markov chain Monte Carlo settings
☒	☐	For hierarchical and complex designs, identification of the appropriate level for tests and full reporting of outcomes
☒	☐	Estimates of effect sizes (e.g. Cohen's *d*, Pearson's *r*), indicating how they were calculated

Our web collection on statistics for biologists contains articles on many of the points above.

Software and code

Policy information about availability of computer code

Data collection	The raw sequencing reads of all libraries are available from EBI/ENA via the project PRJEB52177. WeStCOMS hindcast and forecast modelling data since 2013 are publicly accessible at https://thredds.sams.ac.uk/. Data used to generate all figures are available at https://github.com/pseudogene/Corrochano-Fraile_et_al_2023.
Data analysis	The particle-tracking code can be found at https://github.com/tomadams1982/BioTracker (commit 9fbf1bb). Scripts and workflows used at available at https://github.com/pseudogene/Corrochano-Fraile_et_al_2023.

For manuscripts utilizing custom algorithms or software that are central to the research but not yet described in published literature, software must be made available to editors and reviewers. We strongly encourage code deposition in a community repository (e.g. GitHub). See the Nature Portfolio guidelines for submitting code & software for further information.

Data

Policy information about availability of data

All manuscripts must include a data availability statement. This statement should provide the following information, where applicable:

- Accession codes, unique identifiers, or web links for publicly available datasets
- A description of any restrictions on data availability
- For clinical datasets or third party data, please ensure that the statement adheres to our policy

The raw sequencing reads of all libraries are available from EBI/ENA via the project PRJEB52177.

Research involving human participants, their data, or biological material

Policy information about studies with human participants or human data. See also policy information about sex, gender (identity/presentation), and sexual orientation and race, ethnicity and racism.

Reporting on sex and gender	No human participants
Reporting on race, ethnicity, or other socially relevant groupings	*Please specify the socially constructed or socially relevant categorization variable(s) used in your manuscript and explain why they were used. Please note that such variables should not be used as proxies for other socially constructed/relevant variables (for example, race or ethnicity should not be used as a proxy for socioeconomic status).*<br>*Provide clear definitions of the relevant terms used, how they were provided (by the participants/respondents, the researchers, or third parties), and the method(s) used to classify people into the different categories (e.g. self-report, census or administrative data, social media data, etc.)*<br>*Please provide details about how you controlled for confounding variables in your analyses.*
Population characteristics	*Describe the covariate-relevant population characteristics of the human research participants (e.g. age, genotypic information, past and current diagnosis and treatment categories). If you filled out the behavioural & social sciences study design questions and have nothing to add here, write "See above."*
Recruitment	*Describe how participants were recruited. Outline any potential self-selection bias or other biases that may be present and how these are likely to impact results.*
Ethics oversight	*Identify the organization(s) that approved the study protocol.*

Note that full information on the approval of the study protocol must also be provided in the manuscript.

Field-specific reporting

Please select the one below that is the best fit for your research. If you are not sure, read the appropriate sections before making your selection.

☐ Life sciences ☐ Behavioural & social sciences ☒ Ecological, evolutionary & environmental sciences

For a reference copy of the document with all sections, see nature.com/documents/nr-reporting-summary-flat.pdf

Ecological, evolutionary & environmental sciences study design

All studies must disclose on these points even when the disclosure is negative.

Study description	Blue mussel (Mytilus edulis) connectivity and larvae dispersal. Samples were collected from 13 sites between 2020 and 2021 tot estimate population structure and gene flow. For each site at least 30 samples were collected.
Research sample	Samples of at least 30 mussels were collected from different ropes (if from farm) or rock pools.
Sampling strategy	Power calculation was not appropriate to estimate the samples size necessary, however previous studies () provided evidence that 30 to 40 samples would be enough to be representative of the population allele frequency.
Data collection	Mussel were collect on the shore or from farm ropes, transported to the lab, were they were measured and tissue samples were taken.
Timing and spatial scale	These samples were obtained from natural mussel beds located along the coast in Portree, Applecross Bay, Loch Torridon, Loch na Cairidih, and Bo Sligachan, as well as from mussel farms in Loch Eil, Loch Linnhe, Bàgh a Tuath, Loch Sunart, Loch Spelve, Loch Roag, Loch Laxford, and Badcall Bay between 2020 and 2021 (Full description in Table 1 of the manuscript)
Data exclusions	No data were excluded.
Reproducibility	At those were temporal sampling, no replication was possible. We maximise the number of samples to ensure representativity of the samples.
Randomization	No applicable.
Blinding	Population genetic data, individual results are not analyses, only groups. Analysis was conducted with samples ID and no reference to the sampling origin. The names were added after the analysis to allow interpretation.

Did the study involve field work? ☒ Yes ☐ No

Field work, collection and transport

Field conditions	Variable. As the animals were mostly from farm origin, weather condition were irrelevant.
Location	Multiple locations all stated (with date, longitude and latitude coordinates) in Table S1.
Access & import/export	Animals from farm were handle by farmer and process in the lab. Animals collected on shore were collected under Marine Scotland authority. Animal handling and collection in this study was carried out in accordance with the UK Animals (Scientific Procedures) Act 1986 Amended Regulations (SI 2012/3039).
Disturbance	As DNA was collected disturbance has no impact of the study. Collection on farmed animals was part of the normal harvesting process. Collection on shore animal had minimal impact as only few animals were collected by hand on was mussel beds.

Reporting for specific materials, systems and methods

We require information from authors about some types of materials, experimental systems and methods used in many studies. Here, indicate whether each material, system or method listed is relevant to your study. If you are not sure if a list item applies to your research, read the appropriate section before selecting a response.

Materials & experimental systems

n/a	Involved in the study	
☒	☐	Antibodies
☒	☐	Eukaryotic cell lines
☒	☐	Palaeontology and archaeology
☐	☒	Animals and other organisms
☒	☐	Clinical data
☒	☐	Dual use research of concern
☒	☐	Plants

Methods

n/a	Involved in the study	
☒	☐	ChIP-seq
☒	☐	Flow cytometry
☒	☐	MRI-based neuroimaging

Animals and other research organisms

Policy information about studies involving animals; ARRIVE guidelines recommended for reporting animal research, and Sex and Gender in Research

Laboratory animals	Farm and wild animal only.
Wild animals	Mussel were collected from the sea shore by hand, at random, as much apart as possible. Animal were processed within hours of collection. Sex was not identifiable as Mussel are not sex separated species. Mantle tissue was was collect for DNA analysis and species conformation as in Scottish water, M. edulis, M. galloprovincialis and hybrids are visually indistinguishable.
Reporting on sex	Mussel are not-sex separated species.
Field-collected samples	Farm mussel are collected by farmed and kill on site by on ice. Wild collected animals were transported in cold water and killed on ice in the lab.
Ethics oversight	The work was approved by the University of Stirling Ethics Committee (Animal Welfare and Ethics Review Board).

Note that full information on the approval of the study protocol must also be provided in the manuscript.
